# *De novo* genome sequence of endophytic biocontrol strain *Bacillus* sp. ST24 isolated from rice seed

**DOI:** 10.1128/MRA.00352-23

**Published:** 2023-08-04

**Authors:** Sabin Khanal, Sanjay Antony-Babu, Xin-Gen Zhou

**Affiliations:** 1 Texas A&M AgriLife Research Center, Beaumont, Texas, USA; 2 Department of Plant Pathology and Microbiology, Texas A&M University, College Station, Texas, USA; The University of Arizona, Tucson, Arizona, USA

**Keywords:** *Bacillus*, genomics, *Bacillus cereus*, *Bacillus thuringiensis*, phytobacteriology

## Abstract

In this study, we present the genome sequence of *Bacillus* sp. strain ST24, an endophytic bacterium isolated from rice seeds. The genome assembly comprises a total of 5,799,877 bp, with a GC content of 34.81%. Furthermore, our analysis revealed the presence of various genes associated with antibiotic production, as well as genes involved in polyketide biosynthesis and non-ribosomal polyketide-like clusters.

## ANNOUNCEMENT

*Bacillus* species are endospore-forming aerobic or facultatively anaerobic, Gram-positive bacteria known to be ubiquitous in the environment, with recognized antagonistic ability against diverse phytopathogens (1 - 4). *Bacillus* sp. ST24, an endophytic bacterium, was isolated from rice seeds collected from a research field in Beaumont, Texas. Approximately 20 g of seeds was ground in phosphate-buffered saline (PBS) buffer using a sterile mortar and pestle and plated on tryptic soy agar (TSA). Strain ST24 exhibited strong antagonistic activities against *Rhizoctonia solani* AG11 and *R. solani* AG4, the causative agents of rice seedling blight (5), in our *in vitro* dual-culture plate assays (6).

Genomic DNA extraction was performed using a Zymo Quick Fungal/Bacterial DNA kit following the manufacturer’s instructions, from pure isolated colonies that had been grown on TSA at 27°C for 48 h, suspended in PBS buffer, and adjusted to 10^8^ cells/mL. DNA integrity was evaluated through electrophoresis on a 1% agarose gel. The quality of the DNA was assessed in Quickdrop Micro-volume spectrophotometer (Molecular Devices), while quantification was performed using a Qubit double-stranded DNA high-sensitivity assay kit (Thermo Fisher Scientific). Illumina libraries were prepared using NEB Ultra II DNA Library Prep. Kit and genome sequencing were conducted by Novogene Corporation, Inc., using an Illumina-Novaseq 6000. Strain ST24 generated 9,70,156 paired end reads with insert size of 150 bp in length 220-fold genome coverage. Raw reads below Q30 were trimmed using TrimGalore v0.6.7 (7). High-quality reads were assembled *de novo* using MegaHit v.1.2.8 (8). All assembled reads were assembled into 53 contigs with 5,799,877 total bp. The largest contig and N_50_ value of the assembly were 1,153,785 and 462,236 bp, respectively. The GC content of the assembly was 34.81%. Pilon v. 1.23 (9) and Quast v. 5.0.2 (10) were used to improve and assess the quality of the genome, respectively. BUSCO (11) assessed the genome assembly at 99.2% complete with 95.2% complete and single-copy BUSCOs. Finally, the assembly was annotated using NCBI prokaryotic genome annotation pipeline (12). Genome annotation predicted 5,816 genes with 5,585 protein coding genes, 19 genes encoding rRNA, 45 genes encoding tRNA, 5 ncRNAs, and 1 gene encoding tmRNA. Default parameters were used for all software unless otherwise stated.

The whole 16S rRNA gene of strain ST24 matched 100% with multiple *B. cereus* group through blast on EzTaxon server (13). The average nucleotide identify (ANI) (14) values for strain ST24 were 96.44% and 91.1% when compared to two *B. cereus* strains, ATCC 14579 and strain 30075, respectively. Additionally, the ANI values for strain ST24 were 96.38% and 96.39%, when compared to two *B. thuringensis* strains, ATCC 10792 and MYBT18246, respectively. Furthermore, the Type Genome Server (15) placed strain ST24 in the same species cluster with *B. cereus* ATCC 14579 and *B. thuringensis* ATCC 10792 but with different subspecies cluster.

Through the analysis of the genome of *Bacillus* sp. strain ST24, various genes associated with antimicrobial activities (1, 16, 17), siderophore production (18), phosphate solubilization (19), and 14 biosynthetic clusters (20, 21) were also detected using the antiSmash v.6.0 (22) (Table 1). Collectively, these findings suggest that *Bacillus* sp. strain ST24 holds promise as a potential biocontrol agent for managing seedling blight in rice.

**TABLE 1:** Biosynthetic gene clusters predicted by antiSMASH 6.0 in *Bacillus* sp. strain ST24

Gene cluster	Known cluster	Similarity (%)	Contig number	Nucleotide bp^*a*^	
				From	To
Betalactone	Fengycin	40	28	93,427	118,665
CDPS^*b*^	–^*c*^	–	47	1	15,264
LAP^*d*^	–	–	16	155,987	179,493
NRP^*e*^ + polyketide	Zwittermicin A	81	43	37,965	168,927
NRPS^*f*^	–	–	26	202,102	249,259
	–	–	28	11,431	76,286
	–	–	28	260,001	307,017
NRPS-like	–	–	10	95,846	139,427
RiPP^*g*^-like	–	–	4	155,987	179,493
	–	–	28	169,897	179,357
	–	–	28	234,961	245,209
RRE^*h*^-containing	–	–	43	1	13,099
Siderophore	Petrobactin	100	25	9,617	23,325
Terpene	Molybdenum cofactor	17	33	29,614	51,467

^a^ Start and end nucleotide number for gene cluster.

^*b*^ tRNA-dependent cyclodipeptide synthases.

^*c*^ –, not available.

^*d*^ Linear azol(in)e-containing peptides.

^*e*^ Non-ribosomal peptide.

^*f*^ Non-ribosomal peptide synthetase.

^*g*^ Other unspecified ribosomally synthesized and post-translationally modified peptide product.

^*h*^ RRE-element-containing cluster.

## Data Availability

Data can be assessed under Bioproject number PRJNA880562, with genome assembly at JAOSLI000000000 and raw reads at SRA: SRX17676681.
